# A Unique Case of Cardiac Arrest following K2 Abuse

**DOI:** 10.1155/2014/120607

**Published:** 2014-05-25

**Authors:** Saif Ibrahim, Farah Al-Saffar, Thomas Wannenburg

**Affiliations:** Department of Internal Medicine, University of Florida College of Medicine, 653-1 West 8th Street, Jacksonville, FL 32209, USA

## Abstract

Sudden cardiac death (SCD) accounts for up to 450,000 deaths every year in the United States (Zipes et al. (2006)). Most cases of sudden cardiac death occur in subjects with no prior history of heart disease (Myerburg et al. (1998)). The incidence of sudden death in a general population has been shown to increase contemporaneously with substance abuse (Phillips et al. (1999)). The causative association of sudden death with cocaine, methadone, and volatile agents is well established (Adgey et al. (1995) and Isner et al. (1986)). We describe a case of out-of-hospital cardiac arrest temporally related to abuse of the synthetic cannabinoid street drug known as K2. To our knowledge, there are no previously documented cases of sudden cardiac death associated with synthetic cannabinoids although they have been linked to myocardial infarction in teenagers despite normal coronary angiography (Mir et al. (2011)).

## 1. Introduction

K2, also known as “Spice,” is a synthetic cannabinoid, marketed as incense. It was widely available at specialty shops and on the Internet until new federal legislation classified it as a scheduled 1 controlled substance [1]. Recent reports have noted a steady increase in abuse of synthetic cannabinoids, due to their marijuana-like effects and because they are not detectable on routine urine drug screening [2].

K2 is a herbal blend containing multiple synthetic cannabinoids. The exact composition of this drug is not known and may be variable. The most frequent compound found in the herbal mixture is JWH-018 which was first described by John W Huffman at Clemson University in his efforts to study synthetic analogs of tetrahydrocannabinol (THC) [3, 4]. The cardiovascular effects synthetic cannabinoids are poorly documented.

## 2. Observation

**Figure 1:**
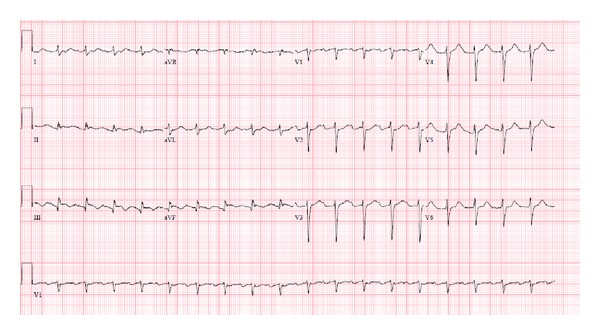
EKG at the time of admission shows sinus tachycardia and inferior myocardial infarction, age undetermined. This EKG also shows a prolonged QTc interval.

A 56-year-old man with a past medical history of hypertension, dyslipidemia, coronary artery disease, and previous myocardial infarction with a four-vessel bypass graft 10 years prior to admission presented to a tertiary care hospital after he suffered a witnessed out-of-hospital cardiac arrest. The patient was witnessed to collapse by a coworker who activated the emergency medical response. Upon arrival of emergency medical service (EMS) the patient was found to be in ventricular fibrillation and cardiopulmonary resuscitation (CPR) was initiated. The patient was defibrillated twice and resuscitation was provided according to the advanced cardiac life support (ACLS) protocol. Epinephrine, atropine, and lidocaine were administered intravenously and within an estimated 15 minutes the patient had a return of spontaneous circulation (ROSC). Upon arrival to the emergency room the patient had a documented pulse which was found to be in sinus tachycardia and remained comatose with a Glasgow coma scale (GCS) of 3. An electrocardiogram (EKG) obtained at the time of admission is shown in (Figure 1).

Upon further history, the family stated that the patient has complained of increasing dyspnea on exertion but no other symptoms including angina were reported. The family described that the patient had recently been using increasing amounts of a street drug called K2 or spice. Laboratory analysis at the time of admission showed elevated troponin T at 0.632 and elevated CKMB (creatinine kinase myocardial band) at 70.2 with an index of 8.6%. These cardiac enzyme levels were the highest as they trended down afterwards. Also noted were the normal levels of potassium, magnesium, and calcium. A routine urine drug screen at the time of admission was negative. He had negative alcohol and salicylate levels. A chest radiograph at the time of admission showed findings suggestive of pulmonary edema. An echocardiogram at the time of admission showed normal left ventricular size and wall thickness and septal asynchrony but no other segmental wall motion abnormality was noted, with an estimated left ventricular ejection fraction (LVEF) of 50%.

Induced therapeutic hypothermia was initiated for neurological protection in the setting of a witnessed ventricular fibrillation arrest with ROSC within 15 minutes. After the patient was successfully rewarmed he had evidence of normal neurological function. He underwent a diagnostic left heart catheterization which showed evidence of severe native coronary vessel artery disease, two saphenous bypass grafts (right coronary artery and first obtuse marginal branch), and a left internal mammary graft to the left anterior artery which were widely patent. A saphenous graft to the first diagonal branch had moderate disease which was not deemed significant. There was evidence of inferior wall hypokinesis on a left ventriculogram. It was felt that he had not suffered an acute coronary occlusion and no “culprit vessel” was identified. Before discharge the patient had an automated implantable cardiac defibrillator (AICD) placed for secondary prevention. A urine screen for Drugs of Abuse was negative on admission. However, the patient admitted to being a long time user of marijuana but recently had been using K2 instead because he could consume larger amounts as it was more affordable. The patient's last consumption was a large amount of K2, right before he “passed out” and was brought to the hospital. He was discharged home on stable condition with meaningful neurological recovery and counseled to abstain for further use of recreational drugs including K2. During follow-up calls made to the patient he mentioned that he quit using K2 and has not had any further cardiac events.

## 3. Discussion

**Figure 2:**
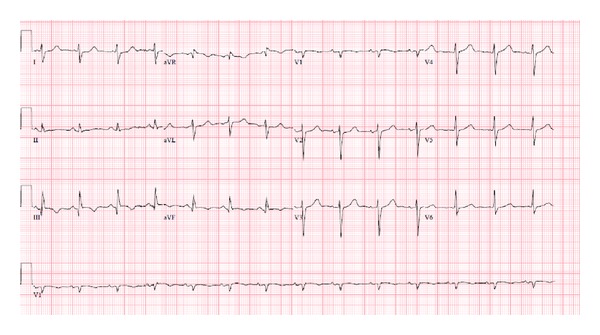
EKG on day 3 of admission shows normal sinus rhythm with significant resolution of QTc prolongation.

Abuse of synthetic cannabinoids has increased among adults as well as teenagers [1, 2]. Our case describes a sudden cardiac arrest within an hour of cannabinoid abuse in a middle-aged male with known coronary artery disease. While preexisting coronary artery disease could have contributed to the patient's presentation, the striking temporal relationship between sudden cardiac arrest and K2 exposure, along with evidence of myocardial necrosis in the absence of an acute coronary occlusion, and no further events with abstinence, all portrait K2 as a causative agent. In support of this hypothesis, there is clear evidence of an arrhythmic effect of marijuana which has been linked to myocardial infarction in patients with normal coronary arteries as well as with sudden cardiac death [5, 6]. This is felt to be mediated by sympathetic nervous stimulation due to release of norepinephrine [7]. It would not be unreasonable to postulate a similar but more potent effect for synthetic cannabinoids, as they too have been found to precipitate myocardial infarction in teenagers in the absence of coronary artery disease [3]. These compounds are agonists of CB1 receptors which is why they initially have a pressor-like effect, followed by vasodilation and subsequent reflex tachycardia and ischemia related to cardiac overload [8–11]. Interestingly though, new studies have suggested that cannabinoids may have an inhibitory effect on myocardial voltage, gated sodium channel, and L-type calcium channels that is independent of their sympathetic action on CB1 and CB2 receptors [12–14]. This might explain the EKG changes seen in this case (Figures 1 and 2). To our knowledge, there are no studies showing specific arrhythmic effects during human clinical electrophysiologic testing.

The risks of cannabinoid exposure may by underestimated as there is no appropriate drug screen. The development of adequate drug screening is complicated by the variability of active compounds in the cannabinoid products. Absent the history from our patient and his family and the evidence of K2 in his possession we may have never had made the link to K2. A sensitive and specific diagnostic screening test is the rate-limiting step in assessing the scope of the problem and the natural history of cannabinoid abuse.

In summary, we present a case of sudden arrhythmic cardiac death in a subject with known coronary artery disease within an hour of exposure to the synthetic cannabinoid marketed as K2. The cardiac arrest was not associated with an acute coronary occlusion but was associated with myocardial necrosis. We postulate that the arrest was triggered by exposure to a synthetic cannabinoid and speculate that this is mediated by sympathetic nervous system activation.
